# Correlates of survival after autoantibody reduction therapy for acute IPF exacerbations

**DOI:** 10.1371/journal.pone.0260345

**Published:** 2021-11-23

**Authors:** Tejaswini Kulkarni, Vincent G. Valentine, Fei Fei, Thi K. Tran-Nguyen, Luisa D. Quesada-Arias, Takudzwa Mkorombindo, Huy P. Pham, Sierra C. Simmons, Kevin G. Dsouza, Tracy Luckhardt, Steven R. Duncan

**Affiliations:** 1 Department of Medicine, University of Alabama at Birmingham, Birmingham, AL, United States of America; 2 Department of Pathology, University of Alabama at Birmingham, Birmingham, AL, United States of America; 3 Department of Medicine, Brigham and Women’s Hospital, Boston, MA, United States of America; 4 Department of Pathology, University of Southern California, Los Angeles, CA, United States of America; 5 Department of Pathology, Michigan Pathology Specialists, Spectrum Health Hospitals, Grand Rapids, MI, United States of America; Helmholtz-Zentrum Munich, GERMANY

## Abstract

**Background:**

No medical treatment has proven efficacy for acute exacerbations of idiopathic pulmonary fibrosis (AE-IPF), and this syndrome has a very high mortality. Based on data indicating humoral autoimmune processes are involved in IPF pathogenesis, we treated AE-IPF patients with an autoantibody reduction regimen of therapeutic plasma exchange, rituximab, and intravenous immunoglobulin. This study aimed to identify clinical and autoantibody determinants associated with survival after autoantibody reduction in AE-IPF.

**Methods:**

Twenty-four(24) AE-IPF patients received the autoantibody reduction regimen. Plasma anti-epithelial autoantibody titers were determined by HEp-2 indirect immunofluorescence assays in 22 patients.

**Results:**

Mean age of the patients was 70 + 7 years old, and 70% were male. Beneficial clinical responses that occurred early during therapy were a favorable prognostic indicator: supplemental O_2_ flows needed to maintain resting S_a_O_2_>92% significantly decreased and/or walk distances increased among all 10 patients who survived for at least one year. Plasma anti-HEp-2 autoantibody titers were ~-three-fold greater in survivors compared to non-survivors (p<0.02). Anti-HEp-2 titers >1:160 were present in 75% of the evaluable one-year survivors, compared to 29% of non-survivors, and 10 of 12 patients (83%) with anti-HEP-2 titers <1:160 died during the observation period (Hazard Ratio = 3.3, 95% Confidence Interval = 1.02–10.6, p = 0.047).

**Conclusions:**

Autoantibody reduction therapy is associated with rapid reduction of supplemental oxygen requirements and/or improved ability to ambulate in many AE-IPF patients. Facile anti-epithelial autoantibody assays may help identify those most likely to benefit from these treatments.

## Introduction

A sizable proportion of patients who have idiopathic pulmonary fibrosis (IPF), estimated as 5–10% annually, develop fulminant exacerbations of their lung disease, not attributable to other causes, that can result in respiratory failure within days [1]. The etiology of these acute exacerbations of IPF (AE-IPF) has been enigmatic, and no medical therapy yet tried has proven efficacy. The short-term mortality of AE-IPF may be as great as 90% or more, depending on disease severity, and these episodes account for ~half of all deaths among IPF patients [1–4].

We, and others, have reported adaptive immune abnormalities that define humoral autoimmune diseases are prevalent in IPF patients [5–18]. Many of these abnormalities are especially prominent among the IPF patients who are having, or will soon have, an acute exacerbation [8, 9, 12, 15, 18]. Moreover, conventional autoantibody syndromes (e.g., connective tissue diseases), can also manifest with sudden lung dysfunction episodes that clinically and histologically mimic AE-IPF. These acute pulmonary exacerbations of antibody-mediated disorders are, like AE-IPF, typically resistant to glucocorticoid-based treatments, but they often respond to modalities that specifically target antibodies [19–24]. We hypothesized similar mechanistically-based therapies might also benefit AE-IPF patients. Encouraging initial results led to the empiric development of a regimen that combined three autoantibody reduction modalities [6]. The present report describes results of our subsequent experiences with these treatments with a particular focus on analyses of patient features and/or laboratory findings that could be associated with patient survival after autoantibody reduction.

## Methods

### Patients

Information was abstracted from prospectively recorded databases of AE-IPF patients who had been admitted to medical and intensive care unit wards at the University of Alabama at Birmingham Hospital (UABH) during the period from May 2016 until August 2018. These patients received the autoantibody reduction regimen as a compassionate use treatment, based on our prior experiences [6]. All patients had been informed these were not standard therapies, and gave verbal consent, in the presence of family members and other medical staff, after being informed about the potential risks, unproven benefit of these modalities, and alternative possible treatments for AE-IPF. None of the subjects were minors, and all had been previously diagnosed with IPF based on contemporary consensus criteria [25]. All fulfilled clinical and radiographic criteria for AE-IPF that included worsening dyspnea and/or hypoxemia within the last 30 days, new infiltrates on chest CT scans superimposed on usual interstitial pneumonia patterns, and exclusions of other causes for their pulmonary dysfunction after detailed evaluations by multiple expert physicians [2]. Studies for respiratory tract bacteria, fungi, and viruses (e.g., microbiological stains, cultures, and serology assays from sputum, blood, and urine) were routine in these patients and were negative in all cases. Other diagnostic testing was based on individual patient assessments by attending physicians. None of these patients had been maintained on immunosuppressants other than prednisone (*<*20 mg/day) before their presentations with AE-IPF, or had recent thoracic procedures, general anesthesia, recognized infections, or other known events that were suspected to have precipitated the exacerbation. None of the patients here were intubated at the time their autoantibody reduction therapies were initiated.

No patient included in this study had clinical or morphological domain features of interstitial pneumonitis with autoimmune features (IPAF) [26] or histories of autoimmune diseases. All had measures of anti-nuclear autoantibodies (ANA), rheumatoid factor (RF) and/or antibodies to citrullinated cyclic peptides (CCP), and most also had assays for anti-Sjögren’s-syndrome-related antigen A (anti-SSA) autoantibodies and myositis panel autoantibodies [27]. Those patients who had positive results on these or other assays of conventional autoantibodies were especially scrutinized for other physical and historical evidence of autoimmune disease symptoms or signs, and these evaluations were negative in all cases.

*Post hoc* analyses of these previously collected data and specimens were approved by the Institutional Review Boards for UABH (#300000944) after the requirement for consent was waived. These analyses were conducted from January 2020 thru June 2021.

### Autoantibody reduction

Patients were treated with a regimen consisting of nine therapeutic plasma exchanges (TPE), two doses of rituximab, and four intravenous immunoglobulin (IVIG) infusions [6], in addition to conventional treatment as usual with steroid and antibiotic therapies (Table 1). Relapses of AE-IPF that occurred after favorable responses to the initial autoantibody reduction course were treated with a modified regimen consisting of five [5] TPE administered every other day, followed by IVIG 0.5 gm/kg/day during each of four successive days.

**Table 1:** Triple-modality autoantibody reduction regimen.

Days on Intervention	1	2	3	4	5	6	7	8	9	10	11	12	13	14	15	16	17	18	19
Prednisone p.o. or i.v. equivalent	60 mg	20 mg each day				none	20 mg each day								none	20 mg each day			
**Therapeutic Plasma Exchange (TPE)**	**x**	**x**	**x**		**x**	**x**			**x**		**x**		**x**		**x**				
**Rituximab**						**1 gm** **IV**									**1gm** **IV**				
**Acetaminophen** (prior to rituximab)						**650 mg Oral**									**650 mg Oral**				
**Solumedrol** (prior to rituximab)						**100 mg** **IV**									**100 mg** **IV**				
**Diphen-hydramine** (prior to rituximab)						**50 mg** **Oral**									**50 mg** **Oral**				
**Empiric Antibiotics**	**x**	**x**	**x**	**x**	**x**	**x**	**x**	**x**											
**IVIG**																**0.5 gm/kg/day**			

**p.o.** = per oral; **i.v.** = intravenous. **Prednisone or i.v., equivalent** were rapidly tapered after the conclusion of this 19 day regimen. **TPE** consist of 1x estimated plasma volume exchanges, replaced with 5% albumin, to maintain a net fluid balance of 95%-100%. The initial three successive TPE are intended to rapidly decrease autoantibody titers in patients with this rapidly progressive syndrome. The 48-hour interruption (days 7–8) enables the first dose of slow-onset rituximab to be administered early, rather than later (e.g., after all TPE), while avoiding removal of the anti-B-cell drug by plasma filtration. When fibrinogen was <120 mg/dL, as may occur in later TPE treatments, either cryoprecipitated antihaemophilic factor (AHF) (two 5-pooled units) or 50% plasma and 50% albumin were given prior to the procedure to raise the fibrinogen level. **Acetaminophen, solumedrol and diphenhydramine** are premedications to obviate reactions to rituximab. **Antibiotics** are standard of practice (SOP) at our institutions for empiric therapy of patients with exacerbations of chronic lung diseases, based on recognition that bronchoalveolar lavage (BAL) have poor diagnostic accuracy and often cannot be safely performed in seriously ill patients. The SOP regimen is: azithromycin (until *Legionella* DFA is negative) plus piperacillin/tazobactam + vancomycin. Substitutions for allergies are ciprofloxacin and linezolid, respectively. Antibiotics are administered as specified by package inserts.

### Clinical measures

Flow rates of supplemental O_2_ necessary to maintain resting S_a_O_2_ >92% immediately prior to beginning treatment (i.e., on day 1 of the regimen) and at the conclusion of the regimen (on day 19) were recorded prospectively. Survival status was documented for all participants by medical records, clinical visits, and telephone interviews. All surviving subjects were observed for >365 days since beginning their AE-IPF treatment. Favorable responses to autoantibody reduction were defined by reductions in the amount of supplemental O_2_ necessary to maintain S_a_O_2_ >92% by >50% and/or increases in walk distance by >30 m (the minimal clinically significant difference [28]) in assessments obtained immediately prior to therapy and on the last day of treatment.

### Laboratory measures

Plasma had been collected during the first TPE from 22 of these patients and was aliquoted and stored frozen (-80°C). These specimens were subsequently used in batched enzyme-linked immunoabsorbant assays (ELISA) for anti-heat shock protein 70 (HSP70) IgG autoantibodies, as previously detailed [8]. These plasma specimens were also used for HEp-2 indirect immunofluorescence assays (IFA), a simple "Gold Standard" for anti-epithelial IgG autoantibody detection and semi-quantitation, as described elsewhere [6, 8, 27]. In brief, commercial slides with fixed, permeabilized HEp-2 [epithelial] cells (Immuno Concepts, Sacramento, CA) were incubated with plasma specimens in serial dilutions from 1:20 to 1:1280, along with autoantibody negative and positive IgG control standards. The plasma was washed off, and replaced with FITC-conjugated anti-human IgG, followed by fluorescence microscopy and imaging. Images were scored by replicate blinded observers to determine the highest (most dilute) titer wherein specimen fluorescence was greater than the concurrent negative IgG controls [6]. These plasma specimens were also used for batched measures of B-cell trophic and chemotactic proteins in a multiplex assay (Milliplex Human Cytokine Kit, MilliporeSigma, Burlington, MA), following the manufacturer’s instructions, i.e., tumor necrosis factor ligand superfamily member 13 (APRIL), chemokine (C-X-C motif) ligand 13 (CXCL13) [15], and B-cell activating factor (BAFF) [18].

### CT measures

In addition to their use in assessments of lung infiltrates [1, 25], chest CT scans were evaluated for the presence of mediastinal lymphadenopathy. Mediastinal lymph nodes (MLN) with short-axis diameter >10 mm were classified as enlarged.

### Statistical analyses

Two group comparisons of continuous or ordered variables were established by Mann-Whitney tests. Comparisons of paired data (e.g., O_2_ flows before and after treatment in individual patients) were made by Wilcoxon. Dichotomous variables were compared by chi-square or Fisher’s exact tests. Survival analyses were performed using product-limit estimation and log-rank tests. Associations between variables were examined by Spearman rank correlation. Hazard ratios (HR) were calculated by proportional hazards and depicted as HR and 95% confidence intervals (CI). Alpha values <0.05 were considered significant. Unless otherwise denoted, data are depicted as means + SD.

## Results

### Patients

**Fig 1:**
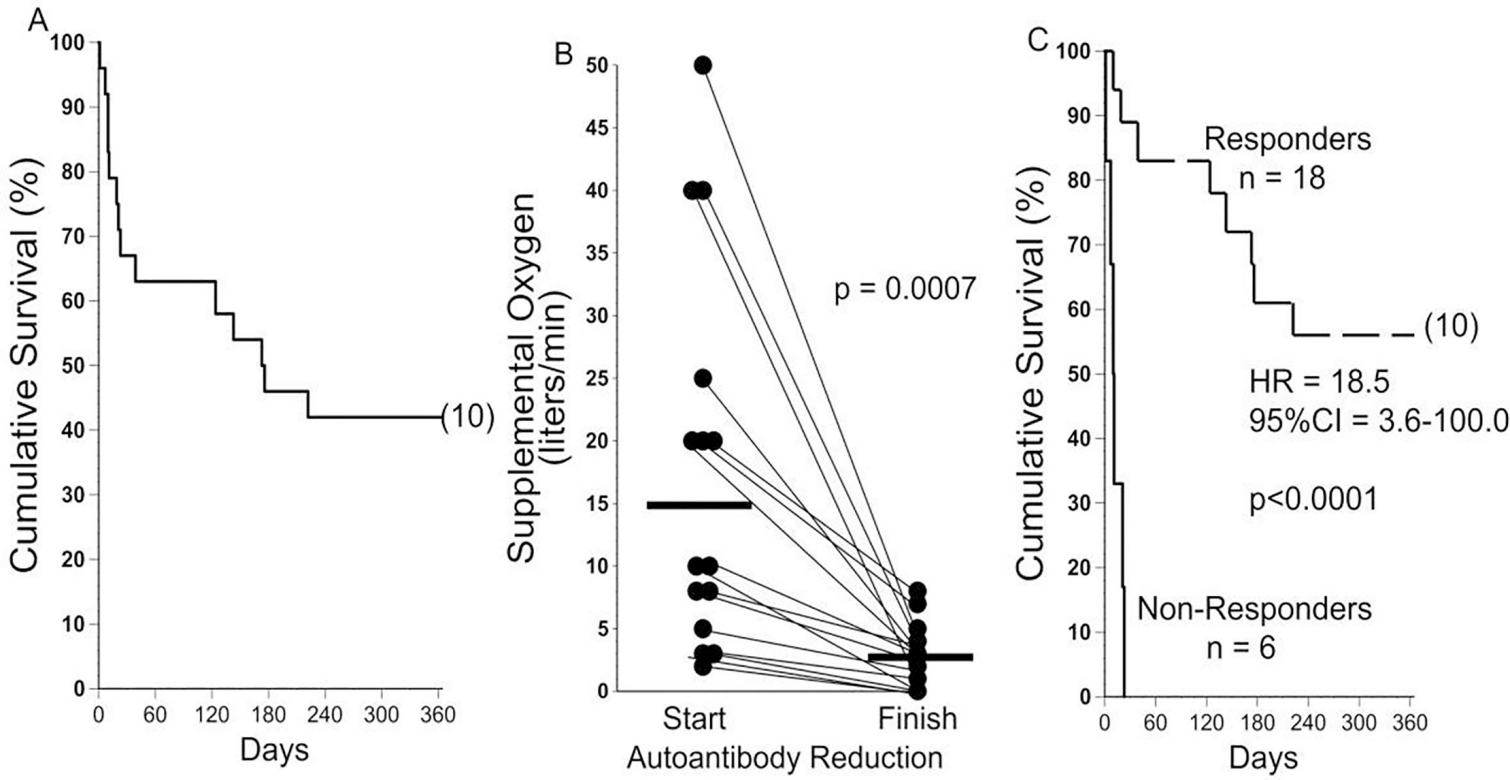
Overall survival and responses to autoantibody reduction therapy. **A)** Cumulative survival of the 24 IPF patients with acute exacerbations. Most of the mortality occurred during the first month after their presentation. Number in parentheses denotes subjects censored at the end of the observation period. **B)** Supplemental oxygen requirements necessary to maintain resting S_a_O_2_ >92% were reduced in 15 AE-IPF patients. Three others (requiring only room air while at rest) increased their six minute walk distances by >30 meter during therapy, and are not shown here. **C)** Survival was prolonged among the 18 AE-IPF who responded to autoantibody reduction therapy with lessened oxygen requirements and/or increased walk distances, in comparison to those patients who had no evident beneficial response to the treatments. HR = hazard ratio; 95%CI = 95% confidence interval. Number in parentheses denotes subjects censored at the end of the observation period.

Characteristics of the AE-IPF patients who underwent autoantibody reduction are detailed in Table 2. Overall one, three, six, and twelve-month survival was 67% + 10%, 63% + 10%, 46% + 10%, 42% + 10% (means and S.E), respectively (Fig 1A).

**Table 2:** Patient demographic characteristics.

	Survivors	Non-survivors	p
Number	10	14	
Age (years)	70 + 8	71 + 7	0.64
Gender (%male)	70	64	0.99
Clinical Laboratory Autoantibodies (%)	50	29	0.40
Chest CT Characteristics			
GGO>50% (%)	40	71	0.21
Honeycombing (%)	90	86	0.99
Lymphadenopathy (%)	80	57	0.39
Any Antifibrotic? (%)	70	57	0.68
Pirfenidone (%)	50	21	0.31
Nintedanib (%)	20	36	0.66
O_2_ Requirement (L/min)	13 + 16	23 + 14	0.064

Characteristics of patients who survived >1 year after initiation of autoantibody reduction (Survivors) vs. those who succumbed within 1 year (non-survivors). Clinical Laboratory Autoantibodies = any patient with any positive test for anti-nuclear autoantibodies (ANA), rheumatoid factor (RF), antibodies to citrullinated cyclic peptides (CCP), anti-Sjögren’s-syndrome-related antigen A (anti-SSA) autoantibodies or myositis panel autoantibodies (S1 Table in S1 File). GGO>50% = ground glass opacities on chest CT involving more than half of the lung fields. All patients had new GGO to some extent.^1^ Lymphadenopathy = >10 mm in short axis.

We could not identify clinical features present at the initiation of therapy that significantly differed between the one-year survivors and non-survivors, although there was a trend for greater initial oxygen requirements among the latter (Table 2). One-year survival was 57% among the 10 patients who required <25 L/min supplemental O_2_ at the start of therapy *vs*. 20% among those with O_2_ prescriptions of >25L/min (p = 0.07). Only one of five patients with an O_2_ requirement of >40L/min at presentation survived one-year (p = 0.36).

O_2_ flow requirements at the start of therapy were significantly less among the 15 patients prescribed an antifibrotic medication (pirfenidone or nintedanib) compared to the nine not taking either of these agents (14L + 14L/min *vs*. 27L + 15L, p = 0.03).

Clinical laboratory assays detected conventional autoantibody abnormalities in near identical proportions of survivors and non-survivors (Table 2) that were, in most cases, barely outside the ranges of normal values (S1 Table in S1 File). There was no predominance of any particular autoantibody specificity in either group (S1 Table in S1 File).

### Responses to autoantibody reduction

Supplemental O_2_ requirements at rest decreased during the 19-day autoantibody reduction therapy in 15 (63%) of these patients, from 15L/min + 15L/min to 3L/min + 3L/min, p = 0.0007) (Fig 1B). Three others (13%) presented with increased requirements for supplemental O_2_ during minimal exertion but were able to maintain their resting S_a_O_2_ >92% on room air. On day 19, after completion of their autoantibody reduction therapy, these three patients had substantial reductions of resting dyspnea and requirements for supplemental O_2_ during ambulation, and all increased their walk distances by >30 m.

Beneficial responses to autoantibody reduction (e.g., lessened supplemental O_2_ requirements and/or increased walk distances, immediately after completion of the therapy) occurred in 13 (87%) of the patients taking an antifibrotic medication, compared to five of those patients not prescribed either of these agents (p = 0.15). There were no significant differences of 12-month survival among those taking or not taking an antifibrotic medication (47% + 13% [SE] *vs*. 33% + 16% [SE], respectively, p = 0.32).

Overall survival was significantly greatest among the 18 patients who evidenced one or both of these favorable responses to the therapy (e.g., better gas exchange and/or increased walk distances) (Fig 1C). Early deaths among at least two of the patients who responded to the therapy with >50% decreases in their O_2_ requirement were due to non-pulmonary atherosclerotic causes (i.e., myocardial infarct, stroke).

The six AE-IPF patients (25%) who did not evidence any beneficial response to autoantibody reduction, but had increasing O_2_ requirements throughout their hospitalizations, (often to unquantifiable flow rates with maximal combined O_2_ delivery systems and/or noninvasive positive pressure ventilation), all suffered early deaths from respiratory failure (12 + 8 days from the start of treatment) (Fig 1C).

Two of the patients who had >50% reductions in supplemental O_2_ requirements during autoantibody reduction again developed features of AE-IPF, without other evident causation or triggers, within three weeks after the conclusion of their first treatment. One of these relapsing patients elected to have an abbreviated treatment regimen for recurrences (five TPE + IVIG). He again had favorable clinical responses per O_2_ requirements and increases in walk distances, and subsequently survived for four more months before dying from recurrent respiratory failure. The other patient declined retreatment and died from progressive respiratory failure within one week.

### HEp-2 and HSP70 autoantibodies, and B-cell mediators

**Fig 2:**
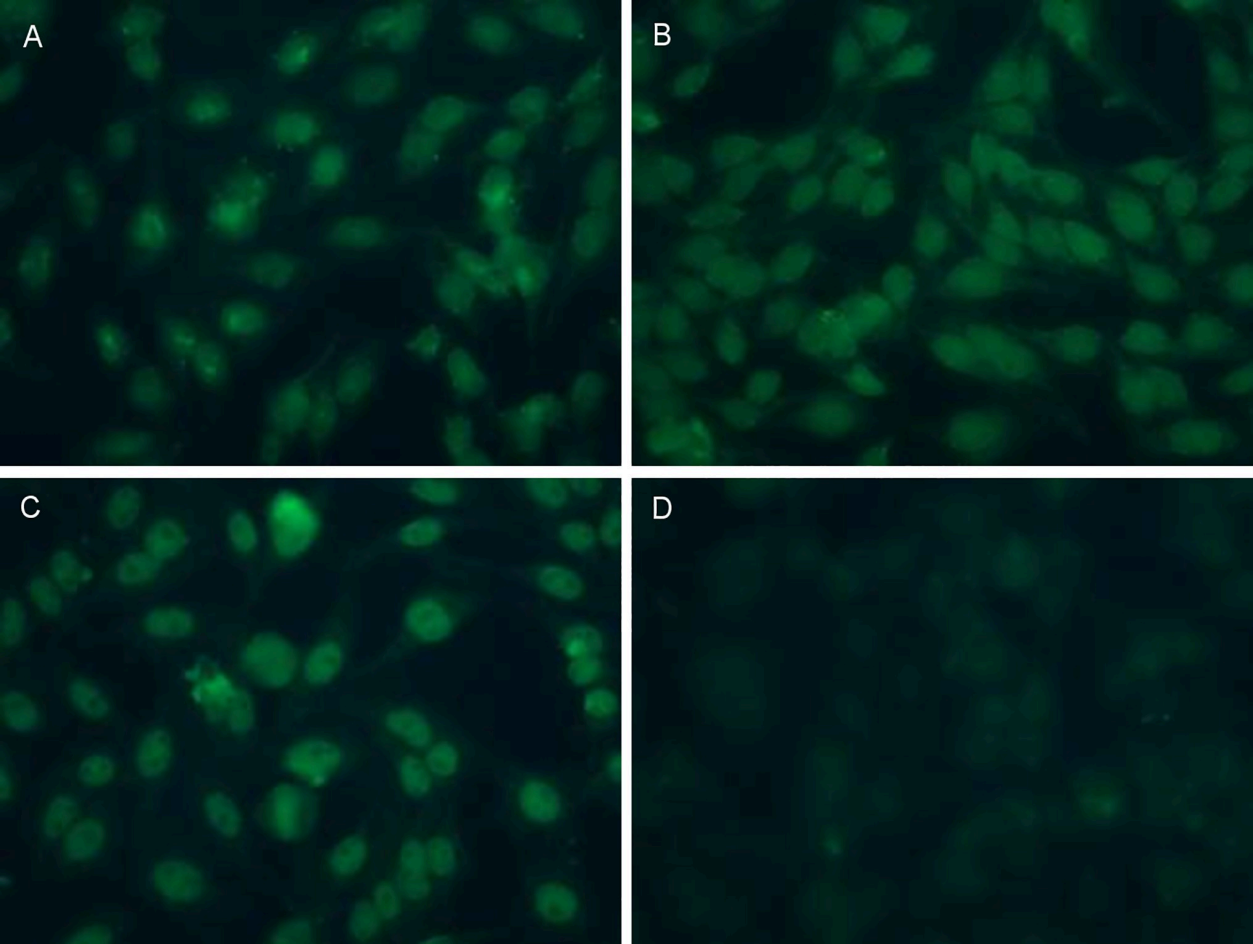
HEp-2 indirect Immunofluorescence (IFA) of IPF Plasma. **A-C)** Illustrate IFA patterns present among AE-IPF patients. All three AE-IPF patients whose plasma HEp-2 images are shown here (A-C) had negative assays for conventional clinical laboratory autoantibodies (see Online S1 Table in S1 File). Titrations here are 1:320 (A), 1:160 (B), and 1:80 (C). **D)** Negative IgG control.

Plasma aliquots from the first TPE were available from 22 of these patients. HEp-2 immunofluorescence staining revealed varied patterns, indicating the presence of autoantibodies with specificities against multiple different epithelial antigens (Fig 2A–2C), in contrast to negative IgG controls (Fig 2D).

**Fig 3:**
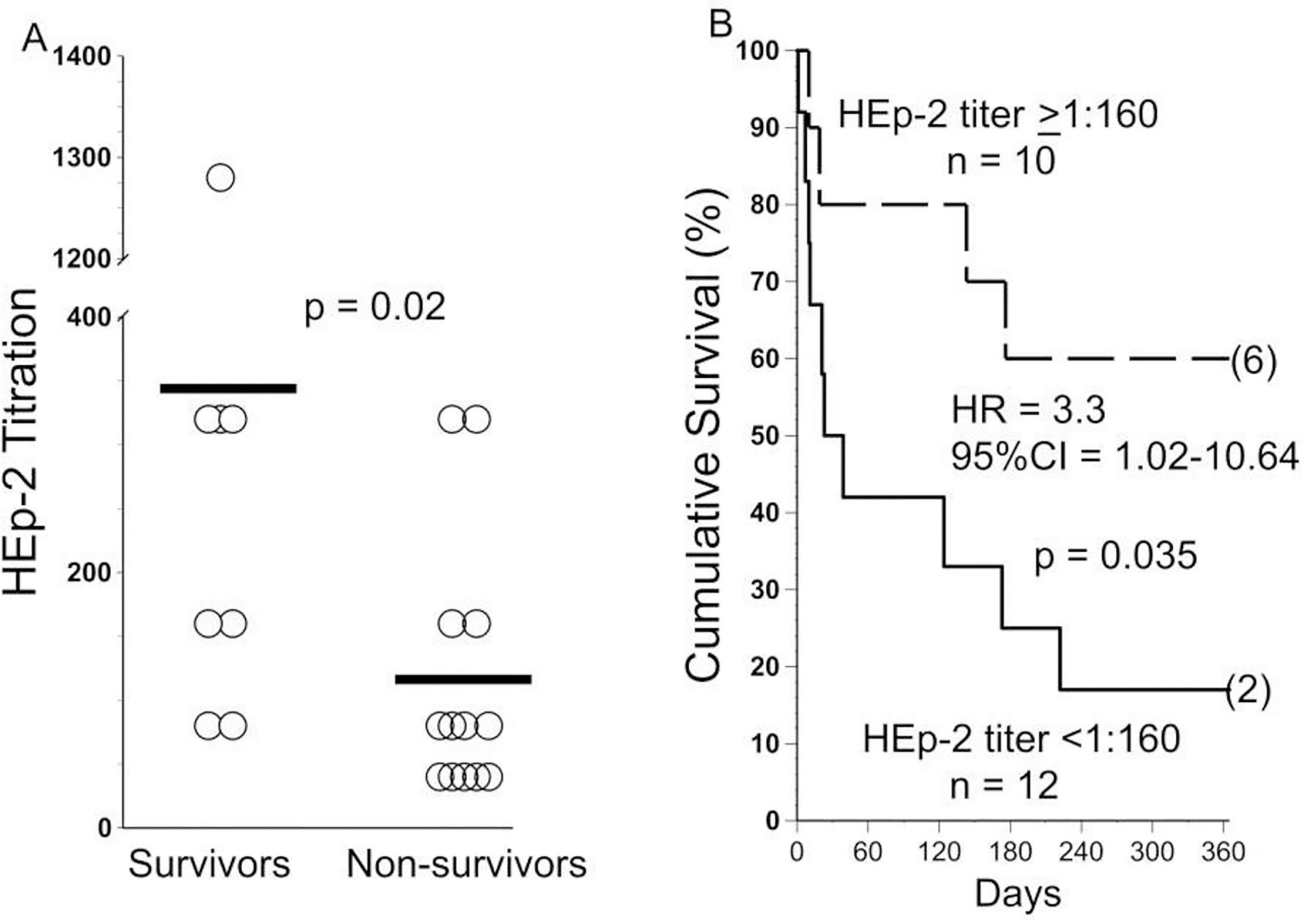
HEp-2 titers and outcomes. **A)** Anti-HEp-2 titers (denoted here as 1:x) were greater among AE-IPF patients who survived for 12 months, compared to those who succumbed. Horizontal lines denote means. **B)** Cumulative survival of subjects with anti-HEp-2 titers >1:160 *vs*. survivals of those with lesser titers. HR = hazard ratio; 95%CI = 95% confidence interval. Number in parentheses denotes subjects censored at the end of the observation period.

Plasma HEp-2 autoantibody titers were higher among the survivors, compared to those who died during the next 12 months (Fig 3A and 3B). Those patients with the highest Hep-2 titers (1:160 and greater) tended to be slightly older, but there were no significant associations of autoantibody titers with gender, antifibrotic use, or initial O_2_ requirement (Table 3).

**Table 3:** Associations with anti-Hep-2 titers at initiation of therapy.

	High Titer anti-Hep-2 (>1:160)	Low Titer anti-HEP-2 (<1:160)	P
n	10	12	N/A
Age (years)	73 + 6	68 + 7	0.057
%Male	70	58	0.67
Antifibrotic Use	60	58	0.99
Supplemental O_2_ need >20L/min at therapy initiation	10	33	0.32

In all but two cases there were complete concordances between replicate HEp-2 titer evaluations by two blinded observers. The discrepant results in these two exceptions were resolved with a tie-breaking assessment by a third blinded referee.

Anti-HSP70 IgG autoantibodies are associated with clinical manifestations and outcome of IPF patients [8], and there was a correlation between plasma concentrations of anti-HSP70 IgG and Hep-2 titers (*r*_*s*_ = 0.49, p = 0.028). Nonetheless, there were no apparent associations of anti-HSP70 concentrations and survival (S1A Fig in S1 File). Similarly, levels of B-cell mediators APRIL, CXCL13, and BAFF did not associate with survival (S1B-S1D Fig in S1 File).

## Discussion

These data show that a triple-modality autoantibody reduction regimen consisting of TPE, rituximab, and IVIG is well-tolerated by AE-IPF patients and seemed to be associated with improved gas exchange in the majority of cases. Although this study does not have a control cohort, the rapid response to these therapies, and cumulative one-year survival (42%) appears to be an improvement compared to published series of responses and outcomes for conventionally-treated AE-IPF patients [1, 3, 4], including our previous compilation of comparable AE-IPF historical controls, in whom one-year survival was zero [6].

We believe these data are consistent with and support findings of several studies that indicate autoimmune mechanisms are involved in the pathogenesis and/or progression of IPF [5–18, 29–31]. Lymphocytes that have been repetitively stimulated by engagements with specific antigens [or autoantigens] undergo pathognomonic differentiation, and abnormally increased proportions of these highly differentiated (and highly injurious) T-cells and B-cells are present in IPF patients [7, 18, 29–31]. Abnormal B-cell infiltrations are often found in lungs of IPF patients, especially among those with more severe and/or acute disease [31–33]. BAFF (*aka* BLyS) is an obligate trophic factor for B-cell survival, maturation, and autoantibody production that is increased in patients with conventional autoantibody disorders [34, 35] and IPF [18] and levels of this mediator are inversely correlated with lung function and survival of the latter. CXCL13 mediates B-cell homing to inflammatory foci and is important in the genesis of immunological diseases [36]. CXCL13 is also over-expressed in AE-IPF patients and, again, levels are inversely correlated with their survival [15]. Many abnormal autoantibodies with diverse avidities have been detected by specialized testing in IPF patients, particularly among those who are having, or soon will have, acute exacerbations, including anti-HSP70 [5, 7–14, 16, 30]. The varied HEp-2 immunofluorescence patterns seen in the present study indicate autoantibodies that bind to several different epithelial cell epitopes were present in the AE-IPF patients, and often in high titers.

IgG autoantibodies can exert pathogenic effects by several mechanisms. Autoantibody-autoantigen aggregates (immune complexes) activate NK cells and complement cascades, which have multiple downstream damaging effects, including neutrophil recruitment, and direct and indirect cytotoxicities [37]. Abnormal immune complexes are present in the circulation [5, 38], bronchoalveolar lavage (BAL) [39], and lung parenchyma of IPF patients [8, 15]. In addition, autoantibodies can also deleteriously affect cell functions by binding to and cross-linking surface receptors that transduce signals. Among other consequences, various target cells that bind unusual autoantibodies found in IPF patients increase their productions of several inflammatory and pro-fibrotic mediators [8, 11–14, 16].

The current treatment regimen (Table 1) is a modification of therapies that have been successfully employed in other autoantibody-mediated diseases and represents the latest iteration in our attempts to develop an efficacious approach to AE-IPF. The foremost component of this regimen is TPE, which rapidly reduces circulating autoantibodies [23, 40]; a necessary treatment effect in these patients whose conditions can deteriorate within days. However, any single plasmapheresis only removes a minor proportion of total body autoantibodies, since concentrations of these immunoglobulins are greatest in extravascular tissues, where they are inaccessible to extracorporeal filtration. Hence, clinically-significant depletion of pathogenic autoantibodies may require multiple TPE over several days, in order to allow interval re-equilibration of the tissue IgG down concentration gradients into the [accessible] plasma compartment [23]. Our initial treatments of AE-IPF with fewer TPE over shorter periods resulted in rapid beneficial responses in several patients, but most of these suffered relapses within days that tended to be quite fulminant and usually lethal [6]. Because TPE effects are short-lived in the absence of additional therapies, rituximab was also added to the regimen to reduce B-cells, the sources for subsequent autoantibody production. However, clinical benefits of rituximab accumulate slowly over weeks to months [41], which precludes its sole use, without prior TPE, in rapidly progressive AE-IPF patients. IVIG has many effects on autoantibody production that include feedback inhibition of residual B-cells by Fc receptor occupancy, and it is often added to TPE and/or rituximab to treat autoimmune diseases [22, 40]. Since adoption of this multi-modality regimen (Table 1) we now observe clinical improvements in more than half of the AE-IPF patients.

The present report has several limitations. The number of available specimens is relatively small so far, which limits the power of assays to identify biomarkers that might distinguish patients who will benefit from autoantibody reduction. The present data are anecdotal results of compassionate-use treatments for an uncommon syndrome with a very high mortality, conducted in a relatively small number of patients, and were not controlled by concurrent comparisons to randomized, conventionally-treated patients. Furthermore, it cannot be ascertained from these data if the early responses seen here in 18 of these patients (75%) were attributable to the TPE or concurrently administered steroids, although the latter seems unlikely given the oft-reported ineffectiveness of those agents in AE-IPF [1, 3, 4, 6]. Nonetheless, these data cannot unequivocally prove that autoantibody reduction is helpful in AE-IPF. A causal relationship between these therapies and improved outcomes is suggested by findings that the beneficial responses were invariably evident very early (i.e., within ~four-to-five days) after starting highly mechanistic treatments, these responses were often of considerable magnitude and durable, clinical improvements were most strongly associated with anti-epithelial autoantibody levels, similar results have now been obtained in several patients treated at different medical centers [6], and an identical beneficial response were seen again among a patient who relapsed and received a second course of autoantibody reduction.

Nonetheless, for the present, autoantibody reduction remains an unconventional and arguably controversial approach in AE-IPF, and unequivocal proof of its efficacy will await results of randomized clinical trials (RCT) with concurrent controls. At least one RCT that could provide more compelling evidence of autoantibody reduction efficacy is now ongoing (*NCT03286556*).

## Conclusions

If treatments that target autoantibodies are shown to be beneficial in AE-IPF, these findings could also conceivably have broader implications in other subpopulations of IPF patients. Incremental trials could further refine and optimize autoantibody reduction regimens, validate measures to facilitate the personalized applications of these treatments, and potentially illuminate other important upstream disease mechanisms aside from or in addition to humoral autoimmunity. Despite the recent introduction of two anti-fibrotic medications, IPF remains a highly morbid and almost invariably fatal disorder [1, 3, 4, 42]. Accordingly, the exploration of novel pathogenic paradigms and potential treatments for this disease seem especially warranted.

## Supporting information

S1 File (DOCX)

S1 Dataset (XLSX)

## References

[1] Collard HR, Moore BB, Flaherty KR, Brown KK, Kaner RJ, King TE, Jr., et al. Acute exacerbations of idiopathic pulmonary fibrosis. Am J Respir Crit Care Med. 2007;176(7):636–43. doi: 10.1164/rccm.200703-463PP 17585107 PMC2094133

[2] Collard HR, Ryerson CJ, Corte TJ, Jenkins G, Kondoh Y, Lederer DJ, et al. Acute Exacerbation of Idiopathic Pulmonary Fibrosis. An International Working Group Report. Am J Respir Crit Care Med. 2016;194(3):265–75. doi: 10.1164/rccm.201604-0801CI 27299520

[3] Mallick S. Outcome of patients with idiopathic pulmonary fibrosis (IPF) ventilated in intensive care unit. Respir Med. 2008;102(10):1355–9. doi: 10.1016/j.rmed.2008.06.003 18635345

[4] Song JW, Hong SB, Lim CM, Koh Y, Kim DS. Acute exacerbation of idiopathic pulmonary fibrosis: incidence, risk factors and outcome. Eur Respir J. 2011;37(2):356–63. doi: 10.1183/09031936.00159709 20595144

[5] Dobashi N, Fujita J, Murota M, Ohtsuki Y, Yamadori I, Yoshinouchi T, et al. Elevation of anti-cytokeratin 18 antibody and circulating cytokeratin 18: anti-cytokeratin 18 antibody immune complexes in sera of patients with idiopathic pulmonary fibrosis. Lung. 2000;178(3):171–9. doi: 10.1007/s004080000020 10871435

[6] Donahoe M, Valentine VG, Chien N, Gibson KF, Raval JS, Saul M, et al. Autoantibody-Targeted Treatments for Acute Exacerbations of Idiopathic Pulmonary Fibrosis. PLoS One. 2015;10(6):e0127771. doi: 10.1371/journal.pone.0127771 26083430 PMC4470587

[7] Feghali-Bostwick CA, Tsai CG, Valentine VG, Kantrow S, Stoner MW, Pilewski JM, et al. Cellular and humoral autoreactivity in idiopathic pulmonary fibrosis. J Immunol. 2007;179(4):2592–9. doi: 10.4049/jimmunol.179.4.2592 17675522

[8] Kahloon RA, Xue J, Bhargava A, Csizmadia E, Otterbein L, Kass DJ, et al. Patients with idiopathic pulmonary fibrosis with antibodies to heat shock protein 70 have poor prognoses. Am J Respir Crit Care Med. 2013;187(7):768–75. doi: 10.1164/rccm.201203-0506OC 23262513 PMC3678112

[9] Kurosu K, Takiguchi Y, Okada O, Yumoto N, Sakao S, Tada Y, et al. Identification of annexin 1 as a novel autoantigen in acute exacerbation of idiopathic pulmonary fibrosis. J Immunol. 2008;181(1):756–67. doi: 10.4049/jimmunol.181.1.756 18566442

[10] Li FJ, Surolia R, Li H, Wang Z, Kulkarni T, Liu G, et al. Autoimmunity to Vimentin Is Associated with Outcomes of Patients with Idiopathic Pulmonary Fibrosis. J Immunol. 2017;199(5):1596–605. doi: 10.4049/jimmunol.1700473 28754682 PMC5563167

[11] Magro CM, Waldman WJ, Knight DA, Allen JN, Nadasdy T, Frambach GE, et al. Idiopathic pulmonary fibrosis related to endothelial injury and antiendothelial cell antibodies. Hum Immunol. 2006;67(4–5):284–97. doi: 10.1016/j.humimm.2006.02.026 16720208

[12] Ogushi F, Tani K, Endo T, Tada H, Kawano T, Asano T, et al. Autoantibodies to IL-1 alpha in sera from rapidly progressive idiopathic pulmonary fibrosis. J Med Invest. 2001;48(3–4):181–9. 11694958

[13] Shum AK, Alimohammadi M, Tan CL, Cheng MH, Metzger TC, Law CS, et al. BPIFB1 is a lung-specific autoantigen associated with interstitial lung disease. Sci Transl Med. 2013;5(206):206ra139. doi: 10.1126/scitranslmed.3006998 24107778 PMC3882146

[14] Taille C, Grootenboer-Mignot S, Boursier C, Michel L, Debray MP, Fagart J, et al. Identification of periplakin as a new target for autoreactivity in idiopathic pulmonary fibrosis. Am J Respir Crit Care Med. 2011;183(6):759–66. doi: 10.1164/rccm.201001-0076OC 20935114

[15] Vuga LJ, Tedrow JR, Pandit KV, Tan J, Kass DJ, Xue J, et al. C-X-C motif chemokine 13 (CXCL13) is a prognostic biomarker of idiopathic pulmonary fibrosis. Am J Respir Crit Care Med. 2014;189(8):966–74. doi: 10.1164/rccm.201309-1592OC 24628285 PMC4098096

[16] Wallace WA, Howie SE. Upregulation of tenascin and TGFbeta production in a type II alveolar epithelial cell line by antibody against a pulmonary auto-antigen. J Pathol. 2001;195(2):251–6. doi: 10.1002/path.916 11592106

[17] Xue J, Gochuico BR, Alawad AS, Feghali-Bostwick CA, Noth I, Nathan SD, et al. The HLA class II Allele DRB1*1501 is over-represented in patients with idiopathic pulmonary fibrosis. PLoS One. 2011;6(2):e14715. doi: 10.1371/journal.pone.0014715 21373184 PMC3044131

[18] Xue J, Kass DJ, Bon J, Vuga L, Tan J, Csizmadia E, et al. Plasma B lymphocyte stimulator and B cell differentiation in idiopathic pulmonary fibrosis patients. J Immunol. 2013;191(5):2089–95. doi: 10.4049/jimmunol.1203476 23872052 PMC3804013

[19] Erickson SB, Kurtz SB, Donadio JV, Jr., Holley KE, Wilson CB, Pineda AA. Use of combined plasmapheresis and immunosuppression in the treatment of Goodpasture’s syndrome. Mayo Clin Proc. 1979;54(11):714–20. 491763

[20] Keir GJ, Maher TM, Hansell DM, Denton CP, Ong VH, Singh S, et al. Severe interstitial lung disease in connective tissue disease: rituximab as rescue therapy. Eur Respir J. 2012;40(3):641–8. doi: 10.1183/09031936.00163911 22282550

[21] Jordan SC, Reinsmoen N, Peng A, Lai CH, Cao K, Villicana R, et al. Advances in diagnosing and managing antibody-mediated rejection. Pediatr Nephrol. 2010;25(10):2035–45; quiz 45–8. doi: 10.1007/s00467-009-1386-4 20077121 PMC2923704

[22] Gelfand EW. Intravenous immune globulin in autoimmune and inflammatory diseases. N Engl J Med. 2012;367(21):2015–25. doi: 10.1056/NEJMra1009433 23171098

[23] Reverberi R, Reverberi L. Removal kinetics of therapeutic apheresis. Blood Transfus. 2007;5(3):164–74. doi: 10.2450/2007.0032-07 19204770 PMC2535895

[24] Dau PC. Immunologic rebound. J Clin Apher. 1995;10(4):210–7. doi: 10.1002/jca.2920100410 8770715

[25] Raghu G, Collard HR, Egan JJ, Martinez FJ, Behr J, Brown KK, et al. An official ATS/ERS/JRS/ALAT statement: idiopathic pulmonary fibrosis: evidence-based guidelines for diagnosis and management. Am J Respir Crit Care Med. 2011;183(6):788–824. doi: 10.1164/rccm.2009-040GL 21471066 PMC5450933

[26] Fischer A, Antoniou KM, Brown KK, Cadranel J, Corte TJ, du Bois RM, et al. An official European Respiratory Society/American Thoracic Society research statement: interstitial pneumonia with autoimmune features. Eur Respir J. 2015;46(4):976–87. doi: 10.1183/13993003.00150-2015 26160873

[27] Solomon DH, Kavanaugh AJ, Schur PH, American College of Rheumatology Ad Hoc Committee on Immunologic Testing G. Evidence-based guidelines for the use of immunologic tests: antinuclear antibody testing. Arthritis Rheum. 2002;47(4):434–44. doi: 10.1002/art.10561 12209492

[28] du Bois RM, Weycker D, Albera C, Bradford WZ, Costabel U, Kartashov A, et al. Six-minute-walk test in idiopathic pulmonary fibrosis: test validation and minimal clinically important difference. Am J Respir Crit Care Med. 2011;183(9):1231–7. doi: 10.1164/rccm.201007-1179OC 21131468

[29] Gilani SR, Vuga LJ, Lindell KO, Gibson KF, Xue J, Kaminski N, et al. CD28 down-regulation on circulating CD4 T-cells is associated with poor prognoses of patients with idiopathic pulmonary fibrosis. PLoS One. 2010;5(1):e8959. doi: 10.1371/journal.pone.0008959 20126467 PMC2813297

[30] Hoyne GF, Elliott H, Mutsaers SE, Prele CM. Idiopathic pulmonary fibrosis and a role for autoimmunity. Immunol Cell Biol. 2017;95(7):577–83. doi: 10.1038/icb.2017.22 28356570

[31] Schiller HB, Mayr CH, Leuschner G, Strunz M, Staab-Weijnitz C, Preisendorfer S, et al. Deep Proteome Profiling Reveals Common Prevalence of MZB1-Positive Plasma B Cells in Human Lung and Skin Fibrosis. Am J Respir Crit Care Med. 2017;196(10):1298–310. doi: 10.1164/rccm.201611-2263OC 28654764 PMC6913086

[32] Parra ER, Kairalla RA, Ribeiro de Carvalho CR, Eher E, Capelozzi VL. Inflammatory cell phenotyping of the pulmonary interstitium in idiopathic interstitial pneumonia. Respiration. 2007;74(2):159–69. doi: 10.1159/000097133 17108669

[33] Balestro E, Calabrese F, Turato G, Lunardi F, Bazzan E, Marulli G, et al. Immune Inflammation and Disease Progression in Idiopathic Pulmonary Fibrosis. PLoS One. 2016;11(5):e0154516. doi: 10.1371/journal.pone.0154516 27159038 PMC4861274

[34] Dillon SR, Harder B, Lewis KB, Moore MD, Liu H, Bukowski TR, et al. B-lymphocyte stimulator/a proliferation-inducing ligand heterotrimers are elevated in the sera of patients with autoimmune disease and are neutralized by atacicept and B-cell maturation antigen-immunoglobulin. Arthritis Res Ther. 2010;12(2):R48. doi: 10.1186/ar2959 20302641 PMC2888197

[35] Francois A, Gombault A, Villeret B, Alsaleh G, Fanny M, Gasse P, et al. B cell activating factor is central to bleomycin- and IL-17-mediated experimental pulmonary fibrosis. J Autoimmun. 2015;56:1–11. doi: 10.1016/j.jaut.2014.08.003 25441030

[36] Rioja I, Hughes FJ, Sharp CH, Warnock LC, Montgomery DS, Akil M, et al. Potential novel biomarkers of disease activity in rheumatoid arthritis patients: CXCL13, CCL23, transforming growth factor alpha, tumor necrosis factor receptor superfamily member 9, and macrophage colony-stimulating factor. Arthritis Rheum. 2008;58(8):2257–67. doi: 10.1002/art.23667 18668547

[37] Mayadas TN, Tsokos GC, Tsuboi N. Mechanisms of immune complex-mediated neutrophil recruitment and tissue injury. Circulation. 2009;120(20):2012–24. doi: 10.1161/CIRCULATIONAHA.108.771170 19917895 PMC2782878

[38] Dreisin RB, Schwarz MI, Theofilopoulos AN, Stanford RE. Circulating immune complexes in the idiopathic interstitial pneumonias. N Engl J Med. 1978;298(7):353–7. doi: 10.1056/NEJM197802162980701 146160

[39] Dall’Aglio PP, Pesci A, Bertorelli G, Brianti E, Scarpa S. Study of immune complexes in bronchoalveolar lavage fluids. Respiration. 1988;54 Suppl 1:36–41. doi: 10.1159/000195495 3231904

[40] Nydegger UE, Sturzenegger T. Treatment of autoimmune disease: synergy between plasma exchange and intravenous immunoglobulins. Ther Apher. 2001;5(3):186–92. 11467755

[41] Emery P, Fleischmann R, Filipowicz-Sosnowska A, Schechtman J, Szczepanski L, Kavanaugh A, et al. The efficacy and safety of rituximab in patients with active rheumatoid arthritis despite methotrexate treatment: results of a phase IIB randomized, double-blind, placebo-controlled, dose-ranging trial. Arthritis Rheum. 2006;54(5):1390–400. doi: 10.1002/art.21778 16649186

[42] Raghu G, Rochwerg B, Zhang Y, Garcia CA, Azuma A, Behr J, et al. An Official ATS/ERS/JRS/ALAT Clinical Practice Guideline: Treatment of Idiopathic Pulmonary Fibrosis. An Update of the 2011 Clinical Practice Guideline. Am J Respir Crit Care Med. 2015;192(2):e3–19. doi: 10.1164/rccm.201506-1063ST 26177183

