# Supplementary material for: Correlates of survival after autoantibody reduction therapy for acute IPF exacerbations
Source: PLoS One. 2021 Nov 23;16(11):e0260345. doi: 10.1371/journal.pone.0260345 (PMC8610261; doi:10.1371/journal.pone.0260345)
Supplement: S1 File — (DOCX) [file pone.0260345.s001.docx]

**ONLINE SUPPLEMENT:**

**Correlates of Survival After Autoantibody Reduction Therapy for Acute IPF Exacerbations**

Tejaswini Kulkarni, M.D.^1^, Vincent G. Valentine, M.D.^1^, Fei Fei, M.D.^2^, Thi K. Tran-Nguyen, B.S.^1^, Luisa D. Quesada-Arias M.D.^3^, Takudzwa Mkorombindo, M.D.^1^, Huy P. Pham, M.D.^4^, Sierra C. Simmons, M.D.^5^, Kevin G. Dsouza, M.B.B.S.^1^, Tracy Luckhardt, M.D.^1^, Steven R. Duncan, M.D.^1^*

Department of Medicine^1^, University of Alabama at Birmingham, Birmingham, AL

Department of Pathology^2^, University of Alabama at Birmingham, Birmingham, AL

Department of Medicine^3^, Brigham and Women's Hospital, Boston, MA

Department of Pathology^4^, University of Southern California, Los Angeles, CA

Department of Pathology^5^, Michigan Pathology Specialists, Spectrum Health Hospitals, Grand Rapids, MI

**Supportive Information Table S1. Clinical Laboratory Autoantibodies Present in the AE-IPF Patients**

|  | **ANA** | **RF/CCP** | **SSA** | **Myositis Panel** | **One-Year Survival?** |
| --- | --- | --- | --- | --- | --- |
|  | <1:80* | RF<1:14*  CCP<19* | <19* | Negative* |  |
| Patient # |  |  |  |  |  |
| 1 | negative | RF-22 | negative | negative | YES |
| 2 | negative | negative | negative | negative | NO |
| 3 | **1:80** | negative | negative | negative | NO |
| 4 | negative | negative | negative | ND | NO |
| 5 | negative | negative | negative | ND | NO |
| 6 | **1:80** | **RF-21** | negative | negative | YES |
| 7 | **1:80** | **RF-34** | negative | negative | YES |
| 8 | negative | negative | negative | negative | NO |
| 9 | negative | **RF-20** | negative | ND | NO |
| 10 | **1:320** | **RF-15** | negative | negative | YES |
| 11 | **1:80** | negative | negative | negative | NO |
| 12 | negative | negative | ND | ND | YES |
| 13 | negative | negative | negative | negative | NO |
| 14 | negative | negative | negative | negative | NO |
| 15 | **1:80** | **CCP-47** | negative | negative | NO |
| 16 | negative | negative | negative | negative | NO |
| 17 | negative | negative | ND | ND | NO |
| 18 | negative | negative | negative | negative | YES |
| 19 | negative | negative | negative | negative | NO |
| 20 | negative | negative | **24** | negative | YES |
| 21 | negative | negative | negative | negative | YES |
| 22 | negative | negative | negative | negative | YES |
| 23 | negative | negative | negative | negative | NO |
| 24 | negative | negative | negative | ND | YES |

*Normal ranges for these autoantibodies. Abnormal (positive) values are listed in **Bold font**. ANA = anti-nuclear autoantibody; RF = rheumatoid factor; CCP = citrullinated cyclic peptides; SSA = anti-Sjögren’s-syndrome-related antigen A (SSA autoantibodies). ND = not done. The myositis panel tests for autoantibody specificities against PL-7, PL-12, EJ, OJ, SRP, MI-2, Fibrillarin (U3 RNP), U2 snRNP, Anti-Jo-1 Ab, TIF1 GAMMA (P155/140), MDA-5 (P140) (CADM-140), NXP-2 (P140), Anti-PM/Scl-100, Anti-U1-RNP, Anti-SS-A 52 kD and is further detailed elsewhere: https://www.mayocliniclabs.com/test-catalog/Clinical+and+ Interpretive/58016composed.

**Supportive Information Figures**

**Supportive Information Figure S1.** These later studies were performed if plasma specimens were available from the AE-IPF patients. None of the measured parameters here appeared associated with patient survival, as evidenced by actuarial analyses comparing survival of patients with >median values (Highest) *vs*. those with values < medians (Lowest). **A)** Anti-heat shock protein 70 (HSP70) IgG autoantibodies (n=21); **B)** tumor necrosis factor ligand superfamily member 13 (APRIL) (n=17); **C)** chemokine (C-X-C motif) ligand 13 (CXCL13) (n=17); and **D)** B-cell activating factor (BAFF) (n=22).
